# An Alternative Approach for Detecting Problematic Alcohol Use: Developing the *Student Alcohol Risk Assessment Scale‐15* Using AI‐Assisted Machine Learning

**DOI:** 10.1002/mpr.70096

**Published:** 2026-07-20

**Authors:** Şükrü Alperen Korkmaz, Pınar Mutlu, Sibel Oymak, Gamze Çan

**Affiliations:** ^1^ Çanakkale Onsekiz Mart University Center for Combating Addiction Application and Research Çanakkale Türkiye; ^2^ Department of Psychiatry Faculty of Medicine Çanakkale Onsekiz Mart University Çanakkale Türkiye; ^3^ Department of Chest Diseases Faculty of Medicine Çanakkale Onsekiz Mart University Çanakkale Türkiye; ^4^ Department of Public Health Faculty of Medicine Çanakkale Onsekiz Mart University Çanakkale Türkiye

**Keywords:** alcohol use disorder, artificial intelligence, logistic regression, machine learning, risky alcohol use, screening

## Abstract

**Background/Aim:**

Risky alcohol use is common among university students and negatively impacts physical and psychosocial health. Current screening instruments focus only on behavioral indicators, neglecting psychosocial factors. This study aimed to develop an AI‐supported, brief student‐focused tool for assessing alcohol‐related risk and to evaluate its preliminary reliability and validity among university students.

**Materials and Methods:**

A total of 599 university students participated in this cross‐sectional study. From a pool of 59 items covering behavioral, psychological, social, and academic questions related to alcohol use, a new risk model was created using 15 items selected with ChatGPT‐supported AI algorithms (SARAS‐15). Risk scores ranged from 0 to 27, categorizing participants into low, medium, and high‐risk groups. The model's performance was evaluated using machine learning methods such as Random Forest, Logistic Regression, SVM, and KNN, along with cross‐validation and ROC analysis. We also analyzed its internal consistency and its correlations with screening instruments (AUDIT, RAPS4‐QF, CAGE).

**Results:**

In machine learning analyses, the logistic regression model achieved the highest performance (93.5% accuracy, F1‐score = 0.864, sensitivity = 0.826, specificity = 0.959). ROC analysis demonstrated excellent discrimination between low‐risk (AUC = 0.96) and high‐risk (AUC = 0.93) groups, with strong discrimination for the intermediate‐risk group (AUC = 0.88). The Random Forest model achieved an overall accuracy of 87%, successfully differentiating between the low‐risk group (F1 score = 0.91) and the high‐risk group (F1 score = 1.00). The new model's Cronbach's alpha was 0.811, with strong convergent validity correlations with screening instruments (AUDIT, *r* = 0.861; RAPS4‐QF, *r* = 0.793; CAGE, *r* = 0.631).

**Conclusion:**

The developed artificial intelligence‐supported 15‐item new risk score model is valid and reliable in assessing risky alcohol use among university students. This scale demonstrates a high level of agreement with traditional tests and can accurately detect three risk levels. Due to its multi‐domain content structure, which addresses both behavioral and psychosocial effects, it serves as a complementary screening tool for early risk identification and intervention planning.

## 1 Introduction

The university years represent a vital period in the lives of young individuals, as they strive to achieve academic success, establish social connections, and continue their personal development. During this period, students may engage in risky behaviors, such as alcohol use, as they acquire new experiences and make independent decisions (Park et al. 2006). According to a study conducted by Davoren et al. (2016), alcohol use is the most prevalent risky behavior among university students. This phenomenon has a significant impact on the physical and psychosocial health, academic performance, and social lives of young individuals (Lorant et al. 2013; Castaño‐Perez and Calderon‐Vallejo 2014; Mekonen et al. 2017). The reasons for the prevalence of alcohol use among university students include social pressures, coping mechanisms for stress, and the desire for independence. Students may turn to alcohol consumption to gain social acceptance, and the freedoms offered by the university environment may encourage this behavior (Wechsler and Nelson 2008). For instance, during social events, alcohol consumption is perceived as a normative behavior, which can lead to increased alcohol use among students (Lewis and Neighbors 2006; O’Grady et al. 2011). This behavior can result in a range of long‐term health problems and addiction risks, as emphasized in a study by Schulenberg et al. (2018). Therefore, screening approaches for university students should not only identify alcohol consumption patterns but also capture the psychosocial and functional difficulties associated with risky use.

Previous research indicates that alcohol consumption is commonly expected among college students, and this trend has remained relatively consistent over recent years (Grucza et al. 2009). A recent study in Europe (2019) found that over half of university students consumed alcohol (Colomer‐Pérez et al. 2019). In a systematic review, hazardous alcohol consumption was observed in two‐thirds of university students in Ireland and the United Kingdom between 2002 and 2014 (Davoren et al. 2016). In a meta‐analysis of studies of university students in Ethiopia, the prevalence of alcohol consumption in the last month was reported to be around 25% (Amare and Getinet 2020). In Türkiye, the prevalence in the previous month was 33%, and the lifetime prevalence was 46% in a systematic review of alcohol use among university students (Orhan et al. 2023). A large‐scale study conducted by (Wechsler et al. 2000) found that approximately 44% of college students in the United States exhibited binge drinking behavior. This study highlights that alcohol consumption, particularly on college campuses, is strongly supported by social norms and widely accepted among students. Davoren et al. (2016) found that although alcohol use among university students decreased in specific periods, it remained at high levels in general. This research, as well as Gambles et al. (2022), found that alcohol consumption among students is strongly related to cultural norms, social acceptance, and stress‐coping strategies. In particular, social events and university culture were identified as essential factors reinforcing alcohol use (Lindman et al. 2000; Hebden et al. 2015). All of these studies suggest that alcohol use among university students continues to be a widespread public health problem, although it varies across societies and cultures. These findings indicate that alcohol use among university students is not only an individual problem but is also related to broader social and environmental factors.

The cultural context of alcohol use in Türkiye should also be considered when interpreting alcohol‐related findings among university students. Drinking patterns in this population may be shaped by social norms, family attitudes, peer‐group expectations, and the degree to which alcohol use is embedded in university social life (Wechsler and Nelson 2008; Lewis and Neighbors 2006; O’Grady et al. 2011; Gambles et al. 2022; Lindman et al. 2000; Hebden et al. 2015). In a systematic review of alcohol use among university students in Türkiye, the reported prevalence of alcohol use was lower than that observed in some Western university samples, suggesting that local cultural and social factors may influence both alcohol exposure and willingness to report alcohol‐related problems (Orhan et al. 2023). These contextual factors may partly explain differences in drinking frequency and alcohol‐related risk indicators across student populations.

Many scales assess alcohol use; some of the most widely used are the Alcohol Use Disorders Identification Test (AUDIT), the Cut‐Down, Annoyed, Guilty, Eye‐Opener questionnaire (CAGE), and the Rapid Alcohol Problem Screen 4–Quantity and Frequency scale (RAPS4‐QF) (Saunders et al. 1993; Cherpitel 2002; O’Brien 2008). Many scales assess alcohol use. Advantages of these scales include their practicality and quick application in clinical settings; they evaluate different dimensions, such as quantity, frequency, loss of control, binge drinking, or alcohol‐related criminality and injury; and they offer cut‐off values for risky alcohol use (Saunders et al. 1993; Cherpitel 2002; O’Brien 2008). These instruments, however, do not address the same clinical or screening target. The CAGE was originally developed to identify alcohol dependence and alcohol related problems, while the AUDIT and RAPS4‐QF were designed to cover a broader spectrum of hazardous, harmful, and problematic drinking (Saunders et al. 1993; Cherpitel 2002; O’Brien 2008; Ewing 1984; Cherpitel 1995). Despite their established value in clinical and research settings, these tools may not fully capture some psychosocial and functional consequences that are particularly relevant to university students, including academic difficulties, financial strain, awareness of help‐seeking options, and perceived associations between alcohol use and emotional symptoms. A multi‐domain, student‐focused risk score could therefore serve as a complementary tool, bringing behavioral indicators together with psychosocial and functional domains that are not always sufficiently represented in conventional screening instruments.

Building on this rationale, the primary objective of the present study was to develop a multi‐domain risk score model for university students that assesses patterns of alcohol use alongside psychosocial, academic, financial, emotional, and awareness‐related indicators. We did not intend this model to replace established screening instruments such as the AUDIT, CAGE, or RAPS4‐QF. Rather, we aimed to examine whether a brief, student‐focused score could offer complementary information for identifying low‐, medium‐, and high‐risk patterns of alcohol use in this population.

## 2 MATERIALS and METHODS

### 2.1 Design and Participants

A cross‐sectional and descriptive study was conducted with university students at xxx, xxx, during the 2023‐2024 academic year (June 2024) and the 2024‐2025 academic year (August and September 2024). The survey, which consisted of 81 questions and was administered online, was accessed via a link provided to students by student representatives and deanships, or by scanning the QR code on a poster describing the study displayed on university boards. Informed consent was obtained from all participants at the beginning of the study, after they were provided with information about the research and asked if they wished to proceed. Only participants who chose “yes” were taken to the questionnaire page, where they could quit at any time. Furthermore, a consent form was provided before the survey to ensure anonymity. The study was open to all students enrolled at the university. Participants did not receive any financial compensation, academic credit, gift, or other incentive for completing the survey. All participants completed the survey within 20–30 minutes. All 599 participants completed the mandatory survey items, and no participants were excluded because of missing data.

The total sample consisted of 599 students enrolled at the university. Sociodemographic information, variables related to alcohol consumption, social and psychological effects of alcohol use, and three widely used scales about alcohol use, which have been validated and are reliable in Turkish, were applied to the participants. Ethics committee approval was received from Çanakkale Onsekiz Mart University Ethics Committee for Non‐Interventional Clinical Trials on 05.06.2024, with approval number 2024/05‐08. This study was conducted in accordance with the guidelines of the Declaration of Helsinki. The study methods were compliant with the STROBE checklist.

### 2.2 Model Development

#### 2.2.1 Demographic Factors

Demographic data such as age, gender, undergraduate department, year of undergraduate study, living arrangement, monthly income, smoking and other substance use, presence of medical and mental illness, medical and mental family history, and family members' alcohol consumption and their opinions about alcohol consumption were recorded as self‐reported 34 questions. The study‐specific questionnaire covered several thematic domains: family and social context, frequency and amount of alcohol use, social and academic/professional functioning, physical and psychological consequences, emotional and mental health‐related perceptions, alcohol‐related risk behaviors, and awareness/intention. Example items included: ‘Do any members of your family consume alcohol?’ for family and social context; ‘How many days per week did you drink alcohol during the last month?’ for frequency of use; ‘How much alcohol do you consume on average at one time?’ for amount of use; ‘Does your alcohol use affect your attendance at classes or work?’ for academic/professional functioning; ‘Have you experienced physical health problems due to alcohol use?’ for physical consequences; ‘Do you think there is a relationship between alcohol use and your depression or anxiety symptoms?’ for emotional and mental health‐related perceptions; ‘Have you ever been in dangerous situations after drinking alcohol, such as accidents, injuries, offences, or unprotected sex?’ for alcohol‐related risk behaviors; and ‘Do you think you should give up alcohol in the future?’ for awareness/intention.

The mean age of the participants was 20.84 ± 3.27 years. Regarding gender distribution, women were overrepresented (66.8%, *n* = 400) compared with men (33.2%, *n* = 199). Most of the participants were first‐year students (*n* = 223, 37.2%), enrolled in medical school (*n* = 255, 42.6%), single (*n* = 588, 98.2%), and lived in dormitories (*n* = 302, 50.4%). Twenty‐three percent of female students (*n* = 92) and 18.6% of male students (*n* = 37) had been previously diagnosed with mental illness. Psychological complaints were present in 25% of the women (*n* = 100) and 23.1% of the men. Medical illness was present in 16.5% of women (*n* = 66) and 14.1% of men (*n* = 28). Family history of mental disorder or alcohol use was similar in both sexes (25% vs. 18.1%; 49.3% vs. 41.7%).

#### 2.2.2 Alcohol Use

It was recorded whether the participants had ever consumed alcohol in their lifetime and whether they had consumed alcohol in the previous year, month, or week. The number of days per week they drank alcohol in the last month (0 days, 1–2 days, 3–4 days, and 5–7 days) and how many standard drinks they consumed (by explaining how the amount of standard drinks was calculated) were determined. The answer to the question “In the last month, have you ever consumed more than five standard drinks (4 standard drinks if you are a woman) in a few hours?” was recorded to screen for binge drinking, also known as heavy episodic drinking (Foxcroft et al. 2015; National Institute of Alcohol Abuse and Alcoholism 2004). Participants were asked when they first started drinking alcohol, with response options including before high school, during high school, within the last year at university, or more than 1 year ago at university. They were also asked with whom they first drank alcohol, with response options including family members, friends, or alone.

#### 2.2.3 Alcohol‐Related Factors

The online survey included a 26‐item section assessing alcohol‐related psychosocial, health, and functional factors. These items were structured as closed‐ended questions rather than open‐ended questions. Response formats varied according to item content and included dichotomous yes/no responses, ordinal frequency or intensity categories, and multiple‐choice categorical options.

Items assessing the presence of a consequence or experience were answered as ‘no’ or ‘yes’. Examples included: ‘Have you ever felt that you needed medical or psychological help because of alcohol use?’ ‘Have you experienced physical health problems due to alcohol use?’ ‘Have you ever been in dangerous situations after drinking alcohol, such as accidents, injuries, offences, or unprotected sex?’ ‘Does your alcohol use affect your attendance at classes or work?’, ‘Do you experience financial difficulties due to alcohol use?’, and ‘Do you think there is a relationship between alcohol use and your depression or anxiety symptoms?’

Items assessing frequency or intensity used ordinal response categories. For example, drinking frequency during the last month was coded as none, 1–2 days/week, 3–4 days/week, or 5–7 days/week, and average drinking quantity was coded according to standard‐drink categories. Other items used multiple‐choice categorical response options when appropriate, such as the context of first alcohol use or perceived effects of alcohol on social life, academic functioning, eating habits, mood, and stress coping.

#### 2.2.4 AI‐Assisted Item Selection

The development of the new risk model followed a stepwise item selection procedure. First, the authors generated an initial pool of candidate items from the study‐specific questionnaire domains, including alcohol consumption patterns, psychosocial and academic/professional consequences, physical and psychological effects, risky situations after drinking, and awareness or intention regarding alcohol use. Conceptually relevant content covered by established alcohol screening instruments, including the AUDIT, CAGE, and RAPS4‐QF, was also considered to ensure adequate coverage of commonly assessed alcohol‐risk indicators. The AI model did not generate the initial item pool; rather, it was used to assist with the structured ranking of author‐generated candidate items.

In total, 72 raw candidate items were compiled. Thirteen items were removed before AI‐assisted scoring because they were either conceptually overlapping with other items or were not suitable for inclusion in an alcohol‐risk score. The latter included items that did not directly assess alcohol‐related behavioral, psychosocial, functional, or awareness‐related risk indicators, were not readily convertible into a numerical score, or primarily captured general background information rather than alcohol‐related risk. After this screening step, 59 unique candidate items remained and were advanced to AI‐assisted scoring.

The 59 candidate items were then scored using the OpenAI ChatGPT‐4.5 API according to six predefined criteria:

– Conceptual relevance to alcohol‐related risk (definition and scope, alignment with theoretical framework, evaluation),
– Including behavioral indicators that overlap with both established screening instruments,
– Reflecting both the individual's awareness, intention to seek help, and psychological affect,
– Taking into account impairments in social and academic functioning,
– Understandability and simplicity (expressions that avoid ambiguity),
– The length of the item should not exceed 20 words.

Each criterion was scored on a 1–5 scale by the AI model, where 1 indicated very low relevance or suitability, and 5 indicated very high relevance or suitability. The total AI‐assisted score was calculated by summing the six criterion scores for each candidate item. This procedure was used only to rank items for preliminary screening and did not replace expert judgment.

The following prompt structure was used for AI‐assisted scoring: “You are assisting with the development of a brief alcohol‐risk assessment tool for university students. Evaluate each candidate item according to the following six criteria: conceptual relevance to alcohol‐related risk, overlap with established alcohol‐risk indicators, relevance to awareness/help‐seeking or motivation for change, coverage of psychosocial or academic/functional impairment, clarity and lack of ambiguity, and brevity. Score each criterion from 1 to 5 and provide a total score. Do not rewrite the item unless the wording is unclear. Return the results in a structured table.” The model parameters were set to temperature = 0.2 and maximum tokens = 256 to reduce output variability.

Participants did not rate the candidate items according to these criteria and were not re‐contacted after AI‐assisted scoring. Their role in the study was to complete the online survey, and their responses were subsequently used for psychometric evaluation and classification analyses. The 20 items with the highest scores were then independently reviewed by four expert authors. Each expert evaluated the candidate items for conceptual relevance to alcohol‐related risk, clinical plausibility, appropriateness for university students, clarity, non‐redundancy, and coverage of behavioral, psychosocial, academic, financial, emotional, and awareness‐related domains. Items were retained when at least 80% agreement among the experts was achieved regarding their suitability for inclusion in the final risk score. This expert review was used as a content and clinical plausibility assessment and should not be interpreted as an independent diagnostic validation procedure. The 15‐item multi‐domain risk score was retained for the final scale. The term multi‐domain refers to the inclusion of alcohol consumption patterns together with psychosocial, academic, financial, emotional, and awareness‐related indicators within a single total risk score. It does not imply that separate latent dimensions or subscales were established in the present study.

The responses to each item were converted into numerical scores as indicated below:

1. Dichotomous (Yes/No) answer questions: No = 0, Yes = 1.
2. Multiple‐choice questions indicating frequency/intensity: For example: ‘How much do you drink on average at a time?’ → 0 = None/1 = 1–2/2 = 3–4/…/4 = 10+
3. Questions based on behavior repetition: For example: ‘After you started drinking, how many times did it happen that you could not stop?’ → score between 0 and 4. Risk‐group thresholds were derived empirically from the observed total‐score distribution in the full sample (*N* = 599). The cut‐off scores of 0–5, 6–12, and 13–27 corresponded approximately to lower‐risk, intermediate‐risk, and upper‐risk ranges in the sample distribution. The expert panel then reviewed these preliminary ranges for clinical plausibility and content consistency with the severity of the endorsed items. In this review, higher risk was interpreted as endorsement of items reflecting greater alcohol‐related consequences, such as academic/work absenteeism, financial difficulties, physical health problems, dangerous situations after drinking, perceived emotional consequences, help‐seeking need, inability to stop drinking, guilt/regret, blackout‐like experiences, or morning drinking. The ranges were also compared conceptually with established alcohol‐risk frameworks, particularly AUDIT‐based low‐risk, hazardous/harmful, and possible dependence risk categories. Because these thresholds were not externally validated against a diagnostic interview or an independent measure of functional impairment, they should be interpreted as preliminary risk categories that require further validation. Finally, a pilot psychometric evaluation was conducted using data from the first 150 student respondents. This evaluation examined internal consistency and item‐total correlations. Cronbach's alpha exceeded 0.80, and all item‐total correlations were above 0.30. Therefore, the 15‐item structure was retained and applied in the full‐sample analyses.

**FIGURE 1:**
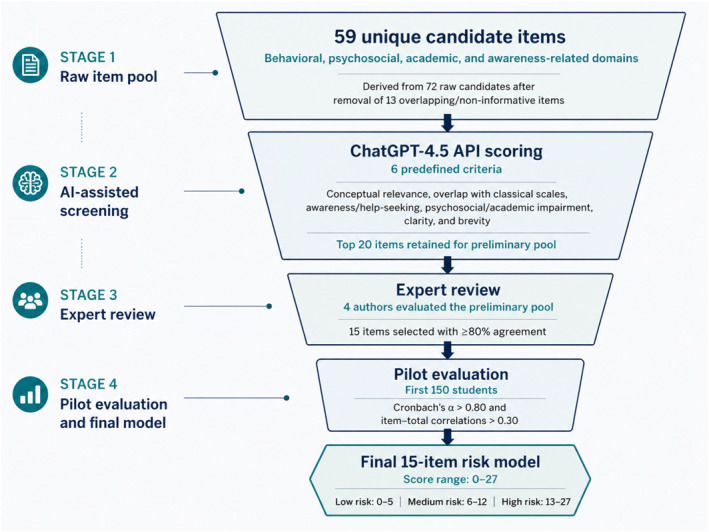
Development workflow of the Student Alcohol Risk Assessment Scale.

Figure 1 illustrates the development process of the 15‐item student‐focused alcohol risk model. The final 15‐item instrument was named the ‘Student Alcohol Risk Assessment Scale‐15’ (SARAS‐15). SARAS‐15 was conceptualized as a new multi‐domain risk model designed to integrate alcohol consumption patterns with psychosocial, academic, financial, emotional, and awareness‐related indicators among university students.

The SARAS‐15 items were revised to use a consistent recent‐risk time frame wherever applicable. Most items refer to alcohol‐related experiences during the past 12 months. This time frame was selected to capture recent and clinically relevant alcohol‐related risk indicators while reducing ambiguity associated with lifetime reporting. Items assessing current awareness or intention were also phrased in relation to thoughts or perceptions experienced during the past 12 months. The only exception was the item assessing drinking frequency during the past month, which was retained to capture recent drinking pattern and to reduce recall bias for frequency estimation.

### 2.3 Measures

The Alcohol Use Disorders Identification Test (AUDIT), CAGE questionnaire, and Rapid Alcohol Problem Screen 4–Quantity and Frequency (RAPS4‐QF) were administered as established screening instruments for alcohol use and alcohol‐related problems. These instruments were used to evaluate convergent validity and to compare the newly developed SARAS‐15 with widely used alcohol screening measures. After electronic informed consent was obtained, the questionnaire was presented in a fixed order. Participants first completed sociodemographic questions, followed by items on alcohol use and consumption patterns, alcohol‐related psychosocial, academic, and functional consequences, and then the established alcohol screening instruments. The screening instruments were administered in the following order: AUDIT, CAGE, and RAPS4‐QF. These instruments were used to evaluate the convergent validity of the SARAS‐15, rather than to assess alcohol consumption frequency alone.

*The AUDIT* consists of 10 questions, with responses to each question scored 0, 1, 2, 3, or 4, except for questions 9 and 10, which allow scores of 0, 2, or 4. The possible total score ranges from 0 to 40. A total score of 0 generally indicates no reported alcohol consumption on the AUDIT items, whereas higher scores reflect increasing levels of alcohol consumption, alcohol‐related harm, or dependence‐related indicators. This alcohol screening instrument comprises three domains: its initial three items gauge alcohol consumption, items 4 through 6 evaluate alcohol dependence, and items 7 through 10 delve into alcohol‐related harm. A score of 1–7 suggests low‐risk alcohol consumption according to the World Health Organization (WHO) guidelines. Scores of 8–14 suggest hazardous or harmful alcohol consumption, while a score of 15 or higher indicates the likelihood of alcohol dependence (moderate‐severe alcohol use disorder). In addition, the Turkish validity study on risky alcohol use proposed a cut‐off value of 8 (Saatçioğlu et al. 2002). Cronbach's alpha for AUDIT was 0.816 in the present study.

*The CAGE* is a screening questionnaire consisting of an acronym of four questions (cut down, annoyed, guilty, eye‐opener) assessing problems related to alcohol use (‘Have you ever felt you should cut down on your drinking?‘, ‘Have people annoyed you by criticizing your drinking?‘, ‘Have you ever felt bad or guilty about your drinking?‘, ‘Have you ever had a drink in the morning to get rid of a hangover (eye‐opener)?’ (Ewing 1984). Each question is scored with a ‘yes (1)’ or ‘no (0)’ response. Higher scores indicate a greater risk for alcohol misuse. Two or more positive responses were used as cut‐off points for identifying alcohol‐related problems with high sensitivity and specificity (Davoren et al. 2016; Gül et al. 2005). The CAGE questionnaire was found to be more effective than other alcohol screening questionnaires in detecting alcohol abuse and dependence. At the same time, AUDIT was superior in identifying at‐risk or harmful drinking (Fiellin et al. 2000). In the present study, the KR‐20 coefficient for the dichotomously scored CAGE questionnaire was 0.530.

*The RAPS4‐QF* is a 6‐item instrument for alcohol dependence, with two additional items added to the RAPS4 (Cherpitel 1995, 2002). The RAPS4 consists of 4 questions about alcohol use: (1) During the last year, have you had a feeling of guilt or remorse after drinking? (Remorse), (2) During the last year, has a friend or family member ever told you about things you said or did while you were drinking that you could not remember? (Amnesia, also called Blackouts), (3) During the last year, have you failed to do what was normally expected of you because of drinking? (Perform), and (4) Do you sometimes take a drink in the morning, when you first get up? (Starter, also called an eye‐opener). The two questions added to RAPS4 in RAPS4‐QF are: (5) During the last year, have you had five or more drinks on at least one occasion? (Quantity) and (6) During the last year, did you drink as often as once a month? (Frequency). A positive response on any of the RAPS4 items (in the first four items) and/or both of the QF items (fifth and sixth items) is considered positive on the RAPS4‐QF. In the present study, the KR‐20 coefficient for the dichotomously scored RAPS4‐QF was 0.696.

The SARAS‐15 was developed as a brief, student‐focused risk assessment tool rather than as a diagnostic instrument. It was not intended to diagnose alcohol use disorder or to screen only for the presence or absence of alcohol use. Instead, SARAS‐15 was designed to classify university students into low‐, medium‐, and high‐risk categories by integrating alcohol consumption patterns with alcohol‐related behavioral, psychosocial, academic, financial, emotional, and awareness‐related indicators. In this manuscript, the term ‘alcohol‐related risk’ refers to patterns and consequences consistent with hazardous, harmful, or problematic alcohol use, without implying a clinical diagnosis of alcohol use disorder.

### 2.4 Data Analysis

We used an AI‐assisted item selection workflow with ChatGPT (OpenAI GPT‐4 API, version 4.5) to rank 59 candidate items based on conceptual relevance, clarity, and brevity. All the items were under 20 words. Prompts were issued via a Python script with a temperature set to 0.2 and a maximum token count of 256. The outputs were fixed by setting a random seed. These parameters were unrelated to model training and were used to ensure that the AI‐assisted item scoring was conducted in a controlled and reproducible manner. A low temperature (0.2) was used to minimize variability in the AI's scoring outputs, ensuring stability across runs. The maximum token count (256) limited the length of the AI's responses to maintain consistent scoring. Setting a random seed ensured that the ranking order of items would remain the same when the script was rerun. In other words, these parameters were used solely to ensure reproducibility and consistency in item scoring, not to influence the test's content. The top 15 items were then reviewed and approved by experts.

All analyses in the study were conducted in a Python 3.10 environment, utilizing open‐source libraries such as Pandas, NumPy, scikit‐learn, SciPy, Matplotlib, and StatsModels. The dataset consisted of 599 participants, all of whom completed the mandatory survey items. No participants were excluded from the analyses. During data preparation, responses were checked for completeness and coding consistency. No missing data were present in the variables included in the analyses; therefore, all analyses were conducted on the full sample without imputation. Continuous variables were summarized as the mean ± standard deviation (SD) in the training set, while categorical variables were presented as frequencies and percentages (%). The data were stratified, with 80% allocated to training and 20% to testing, to preserve the class distribution. Accordingly, the training set included *n* = 480 participants (80%), and the test set included *n* = 119 participants (20%), with stratification preserving the original risk‐group distribution.

During the training phase, continuous features were standardized using the mean and standard deviation (SD) values of the training set; the same transformations were then applied to the test set. For model hyperparameter optimization, 5‐fold stratified cross‐validation and grid search were employed on the training set. The Random Forest algorithm was utilized to classify individuals into ‘Low’, ‘Medium’, or ‘High’ alcohol‐related risk groups based on attributes derived from the 15‐item scale. Hyperparameters were tuned using a stratified 5‐fold grid search: C (Logistic Regression), C and *γ* (SVM with an RBF kernel), *n*_estimators and max_depth (Random Forest), and *k* (KNN). Candidate values were evaluated within predefined ranges, and the configuration that yielded the highest mean cross‐validation macro‐F1 across the training folds was selected. The final models were assessed on a separate test set, and overall accuracy and F1 scores were calculated for each risk class (Low, Medium, High). Differences in performance among the models were analyzed using Friedman's repeated‐measures test, based on results from the test set. The analyses were further supported by the area under the receiver operating characteristic curve (AUC) and a detailed confusion matrix, which assessed each model's robustness to class imbalance and the risk of overfitting. Confusion matrices, accuracy, and F1 scores were computed from each model's predictions on the held‐out test set (20% of the data; *n* = 119).

The SARAS‐15 includes mixed response formats. Most items are dichotomous yes/no items scored as 0 or 1. Items assessing drinking frequency, average drinking quantity, inability to stop drinking after starting to drink, and guilt/regret after drinking are scored using ordinal frequency or intensity categories. Specifically, items 11 and 12 are not dichotomous items: item 11 assesses how many times during the past year the respondent was unable to stop drinking after starting, whereas item 12 assesses how many times during the past year the respondent felt guilty or regretful after drinking. The total score is obtained by summing the item scores, yielding a possible range of 0–27.

Internal consistency was evaluated according to each instrument's response format. For the SARAS‐15, Cronbach's alpha was reported for comparability with previous alcohol‐screening research, and McDonald's omega was calculated as a complementary reliability coefficient for the total risk score because the instrument includes both dichotomous and ordinal items. For the dichotomously scored screening instruments, namely CAGE and RAPS4‐QF, internal consistency was reported using the Kuder–Richardson Formula 20 (KR‐20). KR‐20 is mathematically equivalent to Cronbach's alpha when all items are dichotomously scored. Corrected item‐total correlations were also examined for SARAS‐15 items, with values above 0.30 considered acceptable. The construct validity of the new risk model was evaluated by computing Pearson correlation coefficients between the model's total score and scores from the AUDIT, CAGE, and RAPS4‐QF (convergent validity). To determine the model's generalizability, 10‐fold stratified cross‐validation was conducted using attributes derived from the 15‐item scale.

Permutation importance analysis was used to quantify each item's contribution to the model's predictive performance. The method works by randomly shuffling each feature's values and measuring the resulting decrease in model accuracy. A greater decrease indicates a higher importance of that feature. This approach is model‐agnostic and provides an interpretable measure of each item's contribution to risk classification. We applied permutation importance to identify the most influential items in the 15‐item risk model and to understand the structure of the predictive relationship better. This method is preferable in multi‐domain risk models because it captures contributions that may not be directly reflected in individual model coefficients.

All performance metrics were based on a significance level of *α* = 0.05; however, reliability was assessed using cross‐validation and bootstrapping rather than direct statistical tests for classification metrics. For descriptive proportions, 95% confidence intervals were calculated to characterize the precision of the observed sample‐specific estimates. These proportions were not interpreted as population prevalence estimates because the study did not use a probabilistic sampling design.

## 3 Results

### 3.1 Model Development (AI‐Assisted Machine Learning‐Based Item Selection)

Following the consolidation and expert review described in Section 2.2.4, the final scale contained 15 items. From the items in the pool, 15 items deemed appropriate regarding content similarity, item validity, and measurement scope were selected, and a new model was created (see Table 1).

**TABLE 1:** Student alcohol risk assessment scale (SARAS‐15).

Items	Score range
1. Have you consumed alcohol during the past 12 months?	No = 0, yes = 1
2. How many days a week did you use alcohol in the last month?	None = 0 1–2 days = 1 3–4 days = 2 5+ days = 3
3. How much alcohol do you consume on average at a time (standard drink)?* * 1 standard drink ≈ 10–14 g of pure alcohol (e.g. 1 small beer, 1 glass of wine or 1 single measure of spirits	None = 0 1–2 standard = 1 3–4 standard = 2 5–9 standard = 3 10 + standard = 4
4. Have you ever felt that you needed medical (psychological or physical) help with alcohol use?	No = 0, Yes = 1
5. Do you experience physical health problems due to alcohol use?	No = 0, Yes = 1
6. During the past 12 months, have you been in dangerous situations after drinking alcohol (e.g., accidents, injuries, offences, unprotected sex)?	No = 0, Yes = 1
7. During the past 12 months, has your alcohol use affected your attendance at classes or work?	No = 0, Yes = 1
8. During the past 12 months, have you experienced financial difficulties due to alcohol use?	No = 0, Yes = 1
9. During the past 12 months, have you perceived a relationship between your alcohol use and depression or anxiety symptoms?	No = 0, Yes = 1
10. During the past 12 months, have you thought that you should reduce or stop drinking alcohol?	No = 0, Yes = 1
11. How many times this year have you been unable to stop drinking alcohol after starting to drink? (Binge drinking)	None = 0 1–2 times = 1 3–5 times = 2 6–10 times = 3 10 + times = 4
12. How many times this year have you felt guilty or regretful after drinking alcohol?	None = 0 1–2 times = 1 3–5 times = 2 6–10 times = 3 10 + times = 4
13. During the past 12 months, have you felt that you were doing something bad or wrong because of your drinking?	No = 0, Yes = 1
14. In the last year, has a friend or family member told you about things you have said or done while drinking that you cannot remember (memory loss, loss of consciousness, etc.)?	No = 0, Yes = 1
15. During the past 12 months, have you drunk alcohol first thing in the morning to wake up, feel refreshed, or relieve hangover symptoms?	No = 0, Yes = 1
Total	0–27

The 15‐item alcohol risk model (Student Alcohol Risk Assessment Scale—SARAS‐15) was calculated by converting each item's response to a numerical score and summing the scores. The scoring system was structured as 0–1 (binary) for some items and 0–4 (multiple‐choice) for others. Thus, the theoretical maximum score that can be obtained from the scale is 27. Data distribution was analyzed to convert continuous scores into meaningful risk classes (minimum, median, 75th percentile, and maximum), accounting for both clinical experience and scale similarity. The total points obtained were divided into three risk categories based on the following thresholds: low risk (0–5), medium risk (6–12), and high risk (13–27). The low‐risk range generally reflected no or few endorsed alcohol‐related risk indicators; the medium‐risk range reflected endorsement of several behavioral or psychosocial risk indicators; and the high‐risk range reflected endorsement of more severe or multiple alcohol‐related consequences. These categories should be interpreted as preliminary risk strata rather than diagnostic categories.

The contribution of each feature (question item) in the model to the risk of alcohol use was analyzed using Permutation Importance. In this analysis, the traits with the highest contribution were as follows: Feelings of Guilt/Regret (19.1%, item 12), Average Drinking Amount (14.8%, item 3), Financial Difficulties (14.9%, item 8), and Impact on Physical Health (13.9%, item 5). The effect of gender remained the lowest in the model, at approximately 2.3%.

### 3.2 Creating Risk Categories of the Model

**FIGURE 2:**
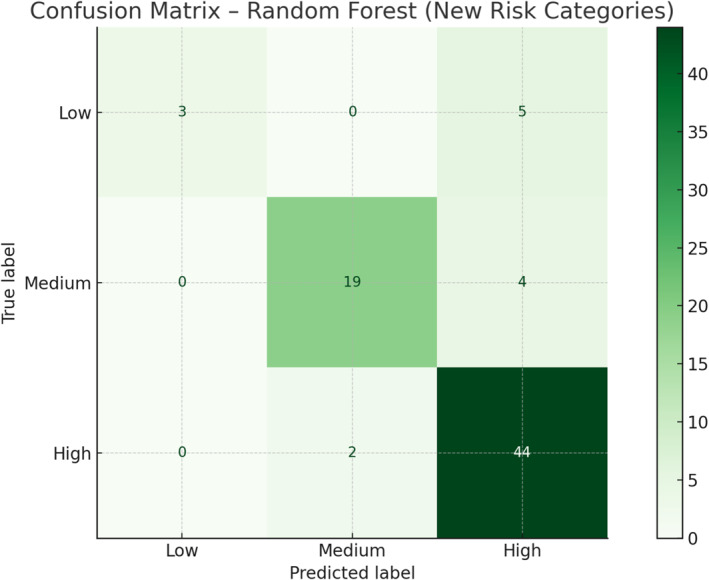
Confusion Matrix showing prediction success according to risk categories. Figure 2 shows the confusion matrix calculated on the independent test set (*n* = 119). The numbers in each cell represent the count of correctly and incorrectly classified cases for the low‐, medium‐, and high‐risk categories. Accuracy (87.0%) was calculated as the proportion of correctly classified test‐set cases out of the total test‐set size. F1 scores reported in the text were derived from class‐specific precision and recall values computed from the same confusion matrix.

In the first classification analysis performed using the Random Forest algorithm, the model achieved an overall accuracy of 87.0% on the test set. Cross‐validation accuracy, averaged across the training folds, was 88.3%, indicating stable model performance. The confusion matrix in Figure 2 reflects the model's performance on the test set (*n* = 119), not on the entire sample. To measure the class‐based discrimination performance, F1 scores for each risk group were calculated: *F*1 = 0.91 for the ‘Low Risk’ group, *F*1 = 0.78 for the ‘Medium Risk’ group, and *F*1 = 1.00 for the ‘High Risk’ group. The model showed the highest classification performance in the low‐ and high‐risk groups (*F*1 = 0.91 and 1.00, respectively), whereas performance was lower in the medium‐risk group (*F*1 = 0.78). This reflects greater overlap in the characteristics of individuals within the medium‐risk category. Overall, the Random Forest approach successfully modeled multidimensional, complex interactions among attributes, achieving reasonable classification performance.

Moreover, various machine learning algorithms were evaluated for classifying individuals' risk levels. The performance of the newly developed classification model was evaluated using three different classifiers: Logistic Regression, Support Vector Machines (SVM), and K‐Nearest Neighbor (KNN). The main performance metrics obtained are presented in Table 2. The Logistic Regression model was particularly notable for its explainability and accuracy. The new risk model was trained and evaluated to categorize individuals into three predefined alcohol‐related risk groups: low, medium, and high. Logistic regression was the most suitable model, both statistically and in practical terms, with high accuracy (93.5%), a micro‐F1 score of 0.888, precision of 0.959, and recall of 0.826. We additionally report macro‐F1 = 0.897, computed as the unweighted mean of class‐specific F1 scores (Low/Medium/High). This indicates that the model showed high precision in classifying participants into the score‐derived SARAS‐15 alcohol‐related risk categories, thereby reducing the likelihood of assigning participants to a higher risk category than their score‐based reference category.

**TABLE 2:** The new risk model's performance table.

Algorithm	Accuracy	F1 (low)	F1 (medium)	F1 (high)
Logistic regression.	93.5%	0.97	0.94	1.00
SVM	90.9%	0.93	0.85	1.00
Random forest	88.3%	0.91	0.78	1.00
KNN	80.5%	0.86	0.59	1.00

Abbreviations: KNN, K‐Nearest Neighbor; SVM, Support Vector Machines.

Multi‐class ROC curves were generated to evaluate the classification performance of the Logistic Regression model. To perform the ROC analysis correctly, only the test data (*y*‐test) was utilized. Due to the multi‐class structure, the actual classes were binarized, and the ‘one‐vs‐rest’ approach was applied for each class. The model's performance was assessed using class‐based AUC (Area Under the Curve) for the redefined triple risk categories: Low (0–5), Medium (6–12), and High (13+). AUC = 0.96 for the Low Risk class; this result indicates that the model has excellent discriminative power in separating the low‐risk group from the other groups. AUC = 0.88 for the Medium Risk class. Because the Medium Risk group shares more characteristics with the other classes, the model's discriminative power is relatively low; however, the AUC remains high, indicating strong classification performance. AUC = 0.93 for the High Risk class: the model accurately discriminates between the high‐risk group, as evidenced by the maximum AUC.

### 3.3 Validity and Reliability Analyses

To examine the predictability of the new risk model scores relative to the established screening instruments—AUDIT, CAGE, and RAPS4‐QF total scores—a random forest regression analysis was performed. The model was built with the Scikit‐learn library, and the dataset was split into 80% for training and 20% for testing. Data from 599 participants were included in the model. The model's overall performance was relatively high. Across the entire sample, the model's explanatory power was *R* ^2^ = 0.994, and the average error was RMSE ≈ 0.28.

However, when the analysis was repeated by gender, a significant decrease in the model's explanatory power was observed. The model's explanatory power for female participants (*n* = 400) was *R* ^2^ = −0.016 and RMSE = 0.72. For male participants (*n* = 199), *R* ^2^ = −0.34 and RMSE = 0.62. The negative *R* ^2^ values in both groups indicate that the model did not provide a statistically significant prediction for gender. These gender‐specific *R* ^2^ results reflect statistical behavior rather than predictive failure: the model reliably predicts alcohol‐risk scores in the combined sample, but the subgroups exhibit limited variance. Rather, they reflect the statistical properties of *R* ^2^ when applied to subsamples with substantially reduced variance. Because *R* ^2^ is calculated relative to the total variance within the subset, homogeneous groups (e.g., males only or females only) may exhibit very low SS_total values. In such cases, even small deviations can produce negative *R* ^2^ values. This does not contradict the overall model performance (*R* ^2^ = 0.994) observed in the full sample, where the variance structure is substantially larger.

For the reliability analysis of the SARAS‐15, internal consistency was examined for the total risk score. Because the SARAS‐15 includes both dichotomous and ordinal response formats, Cronbach's alpha was reported for comparability, and McDonald's omega was calculated as a complementary reliability coefficient. The total SARAS‐15 showed acceptable internal consistency, with Cronbach's alpha = 0.811 and McDonald's omega = 0.831. Corrected item‐total correlations ranged from 0.32 to 0.82, indicating acceptable item discrimination. For the dichotomously scored screening instruments, internal consistency was reported using KR‐20; the KR‐20 coefficients were 0.530 for CAGE and 0.696 for RAPS4‐QF.

The SARAS‐15 total score showed significant positive correlations with all established alcohol screening instruments, supporting convergent validity. The strongest association was observed with AUDIT, followed by RAPS4‐QF and CAGE. Detailed correlation coefficients are presented in Table 3.

**TABLE 3:** Correlations between SARAS‐15 and established alcohol screening instruments.

Screening instruments	SARAS‐15
AUDIT	*r* = 0.861, *p* < 0.001
CAGE	*r* = 0.641, *p* < 0.001
RAPS4‐QF	*r* = 0.793, *p* < 0.001

Abbreviations: AUDIT, Alcohol Use Disorders Identification Test; CAGE, Cut‐Down, Annoyed, Guilty, Eye‐Opener questionnaire; RAPS4‐QF, Rapid Alcohol Problem Screen 4–Quantity and Frequency scale; SARAS‐15, Student Alcohol Risk Assessment Scale‐15.

To evaluate the model's generalizability, 10‐fold stratified cross‐validation was applied. The average accuracy obtained was 97.6% (SD ± 2.2), with the lowest fold accuracy at 94.7%. These findings indicate that the model exhibits consistent generalization performance and does not overfit significantly.

### 3.4 Alcohol‐Related Screening Findings and SARAS‐15 Risk Categories

Alcohol‐related screening findings indicated generally low levels of alcohol‐related risk in the sample, with most participants classified in the low‐risk range according to AUDIT and SARAS‐15. Men had higher mean AUDIT and SARAS‐15 scores than women and were more frequently in the medium SARAS‐15 risk category. In contrast, CAGE and RAPS4‐QF screening positivity rates did not differ significantly by sex/gender. Detailed proportions for each screening instrument and SARAS‐15 risk category are presented in Table 4.

**TABLE 4:** Alcohol‐related screening findings and SARAS‐15 risk categories in the study sample.

Instruments	Proportions in the study sample
AUDIT	5.8% (95% CI, 4.2%–8.0%) was harmful consumption, and 2.5% (95% CI, 1.5%–4.1%) was dependent. Harmful consumption was more observed in men (4.0% vs. 9.5%). Proportion with possible dependence‐related risk were similar between genders, with 1.75% for females and 4% for males.
CAGE	10.4% (95% CI, 8.2%–13.0%) alcohol‐related problems. No significant difference between genders.
RAPS4‐QF	The positive screening rate of alcohol‐related problems is 30.2% (95% CI, 26.7%–34.0%). No significant difference between genders.
SARAS‐15	22.9% (95% CI, 19.7%–26.4%) were in the moderate‐risk group and 2.5% (95% CI, 1.4%–4.1%) in the severe‐risk group. The proportion of men was higher in the moderate‐risk category (19.5% vs. 29.6%); the proportion in the high‐risk category was similar across genders (2.5% in both).

Abbreviations: AUDIT, Alcohol Use Disorders Identification Test; CAGE, Cut‐Down, Annoyed, Guilty, Eye‐Opener questionnaire; CI, confidence interval; RAPS4‐QF, Rapid Alcohol Problem Screen 4–Quantity and Frequency scale; SARAS‐15, Student Alcohol Risk Assessment Scale‐15.

According to the SARAS‐15, the mean score was 3.65 ± 3.64. The mean score of male students (4.37 ± 3.75) was significantly higher than that of female students (3.27 ± 3.54) (*p* < 0.001). According to this model, 74.6% (*n* = 447) of the students were classified as low risk, 22.9% (*n* = 137) as moderate risk, and 2.5% (*n* = 15) as high risk. Similar to AUDIT, the proportion of women was more pronounced in the low‐risk category (78.0% vs. 67.8%), whereas the proportion of men was higher in the moderate‐risk category (19.5% vs. 29.6%, *p* = 0.02); the proportion in the high‐risk category was similar (2.5% percent in both genders). According to the risk categories, there was no significant difference regarding age, grade repetition, parental education level, current psychiatric treatment, and family history of mental illness (all *p* > 0.05). It was found that individuals classified in the medium and high‐risk categories had more psychological complaints, smoked more cigarettes, and had more family members who consumed alcohol (all *p* < 0.001). Moreover, the rate of binge drinking in the last month was higher in the high‐risk group (80%) than in the low‐risk group (4.9%) (*p* < 0.001). Compared to the low‐risk group, those in the high‐risk group had higher rates of negative impacts on academic performance due to alcohol, experiencing alcohol‐related medical illnesses and needing help, encountering dangerous situations or legal issues related to alcohol, being unable to attend classes, missing assignments or exams, neglecting professional responsibilities, facing financial difficulties, experiencing sleep disorders, experiencing alcohol‐related mood changes, and perceiving alcohol as a serious problem (all *p* < 0.05). See Table 4.

When the questions in the model were analyzed (e.g., question 2), 45.0% (95% CI, 41.1%–49.1%) of participants reported consuming alcohol in the past month, with 9.8% (95% CI, 7.7%–12.5%) of the students having consumed alcohol three or more days a week during the same period, a higher proportion of male students reported this behavior (17.1% vs. 6.0%, *p* < 0.001). 6.7% (95% CI, 4.9%–9.0%) of the students consumed five or more standard drinks at a time (alcohol bingeing, 14.1% of males and 3.5% of females, *p* < 0.001). However, only 2.2% of the students reported needing medical assistance. Overall, 8.5% (95% CI, 6.5%–11.0%) of participants reported that their own alcohol use affected their attendance at classes or work, 10.5% (95% CI, 8.3%–13.2%) reported experiencing financial difficulties due to their own alcohol use, and 15.5% (95% CI, 12.8%–18.6%) reported perceiving a relationship between their alcohol use and depression or anxiety symptoms.

## 4 Discussion

The present study developed and preliminarily evaluated SARAS‐15, a brief, student‐focused, multi‐domain risk score designed to assess alcohol‐related risk among university students. SARAS‐15 is not intended to diagnose alcohol use disorder or to screen solely for the presence or absence of alcohol use. Rather, it aims to classify students into score‐derived alcohol‐related risk categories that reflect hazardous, harmful, or problematic patterns of alcohol use, along with alcohol‐related psychosocial, academic, financial, emotional, and awareness‐related indicators. The findings provide preliminary support for its internal consistency, convergence with established alcohol screening instruments, and its ability to classify participants into low‐, medium‐, and high‐alcohol‐related risk categories.

The SARAS‐15 was designed to integrate multiple student‐relevant content domains into a single alcohol‐related risk score. Rather than focusing solely on alcohol consumption frequency or quantity, the item set also includes indicators of difficulty stopping drinking after initiation, alcohol‐related physical and emotional consequences, academic and work attendance difficulties, financial problems, dangerous situations after drinking, perceived need for help, guilt and regret, blackout‐like experiences, and morning drinking. In this respect, SARAS‐15 may provide a broader description of alcohol‐related risk among university students while remaining brief enough for use in research and screening contexts. In response to the need for temporal consistency, the SARAS‐15 items were standardized to assess alcohol‐related risk indicators primarily within the past 12 months, while the past‐month drinking frequency item was retained as an indicator of recent consumption pattern.

The classification findings suggest that SARAS‐15 can distinguish score‐derived alcohol‐related risk categories with acceptable performance. The low‐ and high‐risk categories were classified more accurately than the medium‐risk category, as expected, because intermediate‐risk students may be more heterogeneous. Students in this range may endorse varying combinations of alcohol consumption, psychosocial consequences, academic or financial difficulties, and awareness‐related indicators. Therefore, the medium‐risk category should be interpreted as a preliminary risk stratum that may require further assessment rather than as a distinct clinical group. Similarly, the high‐risk category should not be interpreted as indicating a diagnosis of alcohol use disorder, but rather as reflecting a higher burden of alcohol‐related risk indicators that may warrant further assessment, brief counseling, or preventive support. In practical terms, the low SARAS‐15 risk category reflects no or few endorsed alcohol‐related risk indicators, the medium category reflects several behavioral or psychosocial indicators, and the high category reflects multiple or more severe alcohol‐related consequences. These categories are intended to guide risk stratification and further assessment rather than to indicate alcohol use disorder diagnoses.

The pattern of correlations with established alcohol screening instruments supports the convergent validity of SARAS‐15. The strongest association was observed with AUDIT, which is consistent with the broader content coverage of AUDIT, including alcohol consumption, dependence‐related indicators, and alcohol‐related harm. The association with RAPS4‐QF was also strong, suggesting overlap between SARAS‐15 and established indicators of alcohol‐related problems. The comparatively lower association with CAGE is clinically plausible, given that CAGE is a brief instrument focused primarily on dependence‐related indicators and does not assess drinking frequency or quantity. Thus, SARAS‐15 appears to capture alcohol‐related risk indicators that overlap with established screening instruments while also incorporating student‐relevant psychosocial and functional content.

Several limitations should be considered when interpreting these findings. First, the study used a cross‐sectional design and a non‐probabilistic sample from a single university; therefore, the observed alcohol‐related screening rates should not be interpreted as population prevalence estimates. Second, the SARAS‐15 risk categories were derived from the observed score distribution and reviewed for clinical plausibility, but they were not externally validated against a structured diagnostic interview, an independent functional impairment measure, or prospectively assessed alcohol‐related outcomes. Third, although SARAS‐15 was designed to cover multiple alcohol‐related content domains, its factorial structure was not formally examined in the present study; therefore, the findings support its use as a total multi‐domain alcohol‐related risk score rather than as a set of empirically established subscales. Fourth, the AI‐assisted component was used only as a structured item‐ranking procedure applied to author‐generated candidate items and should not be interpreted as a replacement for expert review or conventional psychometric validation. Finally, the relatively low AUDIT scores and the specific cultural context of alcohol use in Türkiye may limit the generalizability of the findings to populations with different drinking norms, alcohol availability, or campus drinking cultures. Future studies should examine the factorial structure, test–retest reliability, measurement invariance, diagnostic accuracy, and external validity of SARAS‐15 in independent, culturally diverse, and clinical samples.

In conclusion, SARAS‐15 is a brief, student‐focused, multi‐domain risk score designed to assess alcohol‐related risk among university students. The present findings provide preliminary support for its internal consistency, convergence with established alcohol screening instruments, and ability to classify participants into score‐derived alcohol‐related risk categories. SARAS‐15 should not be interpreted as a diagnostic instrument for alcohol use disorder or as a replacement for established screening instruments. Rather, it may serve as a complementary tool for identifying students who may benefit from further assessment, brief counseling, or targeted preventive support. Further validation studies in independent, culturally diverse, and clinical samples are required before SARAS‐15 can be recommended for broader use in research or practice.

## Author Contributions

**Şükrü Alperen Korkmaz:** conceptualization, methodology, resources, investigation, data curation, formal analysis, writing – original draft, writing – review and editing. **Pınar Mutlu:** conceptualization, methodology, resources, investigation, data curation, writing – original draft, writing – review and editing. **Sibel Oymak:** conceptualization, resources, investigation, data curation, formal analysis, writing – original draft, writing – review and editing. **Gamze Çan:** conceptualization, investigation, data curation, supervision, writing – original draft, writing – review and editing.

## Funding Statement

The authors have nothing to report.

## Ethics Statement

The Non‐interventional Ethics Committee of Çanakkale Onsekiz Mart University, Türkiye, approved on 05.06.2024, with approval number 2024/05‐08.

## Consent

All participants were informed about the study, and written consent was obtained before participation in the survey.

## Conflicts of Interest

The authors declare no conflicts of interest.

## Data Availability

The data that support the findings of this study are available from the corresponding author upon reasonable request. The availability of these data is restricted due to ethical and privacy considerations.
